# Chinese herbal medicine for the treatment of primary hypertension: a methodology overview of systematic reviews

**DOI:** 10.1186/s13643-016-0353-y

**Published:** 2016-10-20

**Authors:** Zhao Xinke, Li Yingdong, Feng Mingxia, Liu Kai, Chen Kaibing, Lu Yuqing, Sun Shaobo, Song Peng, Liu Bin

**Affiliations:** 1 School of Basic Medical Sciences, Lanzhou University, Lanzhou, 730000 China; 2 Key Lab of Prevention and Treatment for Chronic Disease by Traditional Chinese Medicine of Gansu Province, Lanzhou, 730000 China; 3 The Hospital Affiliated to Gansu College of TCM, Lanzhou, 730020 China

**Keywords:** Chinese herbal medicine, Primary hypertension, Overview, GRADE, Quality of evidence, Methodological quality

## Abstract

**Background:**

Chinese herbal medicine has been used to treat hypertension in China and East Asia since centuries. In this study, we conduct an overview of systematic reviews of Chinese herbal medicine in the treatment of primary hypertension to 1) summarize the conclusions of these reviews, 2) evaluate the methodological quality of these reviews, and 3) rate the confidence in the effect on each outcome.

**Methods:**

We comprehensively searched six databases to retrieve systematic reviews of Chinese herbal medicine for primary hypertension from inception to December 31, 2015. We used AMSTAR to evaluate the methodological quality of included reviews, and we classified the quality of evidence for each outcome in included reviews using the GRADE approach.

**Results:**

A total of 12 systematic reviews with 31 outcomes were included, among which 11 systematic reviews focus on the therapeutic effect of Chinese herbal medicine combined with conventional medicine or simple Chinese herbal medicine versus simple conventional medicine. Among the 11 items of AMSTAR, the lowest quality was “providing a priori design” item, none review conformed to this item, the next was “stating the conflict of interest” item, only three reviews conformed to this item. Five reviews scored less than seven in AMSTAR, which means that the overall methodological quality was fairly poor. For GRADE, of the 31 outcomes, the quality of evidence was high in none (0 %), moderate in three (10 %), low in 19 (61 %), and very low in nine (29 %). Of the five downgrading factors, risk of bias (100 %) was the most common downgrading factor in the included reviews, followed by imprecision (42 %), inconsistency (39 %), publication bias (39 %), and indirectness (0 %).

**Conclusions:**

The methodological quality of systematic reviews about Chinese herbal medicine for primary hypertension is fairly poor, and the quality of evidence level is low. Physicians should be cautious when applying the interventions in these reviews for primary hypertension patients in clinical practice.

**Electronic supplementary material:**

The online version of this article (doi:10.1186/s13643-016-0353-y) contains supplementary material, which is available to authorized users.

## Background

Primary hypertension is associated with structural changes of the heart and blood vessels, which may lead to cardiovascular morbidity (i.e., cardiovascular disease, stroke, peripheral vascular disease, renal disease, and Alzheimer’s) and mortality. However, the pathogenesis of primary hypertension remains unclear at present [1]. Primary hypertension is typically defined as having a systolic blood pressure (SBP) ≥140 mmHg and a diastolic blood pressure (DBP) ≥90 mmHg [2, 3]. Globally, approximately one billion people are affected by primary hypertension [2], and seven million deaths per year may be related to primary hypertension [4]. In addition, for every 20 mmHg increase in SBP and 10 mmHg increase in DBP (through the range from 115/75 to 185/115 mmHg) among people aged 40 to 70 years, the risk of cardiovascular disease (CVD) morbidity doubles [2].

The current practice focuses on achieving a target blood pressure level less than 140/90, which is believed to be helpful in reducing the risk of stroke and myocardial infarction and improving quality of life. However, while hypertension contributes to adverse cardiovascular outcomes, lowering blood pressure to below this arbitrary value has not been convincingly shown to reduce cardiovascular morbidity and mortality [5]. This finding highlights the importance of finding safe and effective treatments to prevent hypertension-related mortality and morbidity.

The ultimate aim of treating hypertension is to reduce morbidity and mortality with minimum adverse effects. Diuretics, beta-blockers, calcium-channel blockers, and angiotensin-converting enzyme (ACE) inhibitors are commonly used as antihypertensive drugs [6–8]. Although many different antihypertensive drugs are available, the BP levels of approximately two thirds of the patients under treatment have not reached the target level [3, 9]. Even if blood pressure has been controlled within a normal range, patients may still have high cardiovascular morbidity and mortality rates [10].

Chinese herbal medicine has been used to treat hypertension in China and East Asia since centuries. It usually applies a combination of several (often more than 10) herbs that make up a formula under the guidance of traditional theory. Understanding the effect of Chinese herbal medicine on blood pressure could be valuable for the management of high blood pressure. Currently, there are several systematic reviews published regarding the effect of Chinese herbal medicine on primary hypertension, which indicated that Chinese herbal medicines (e.g., the liuwei dihuang pill and tianma gouteng yin) were effective and safe for primary hypertension when compared with conventional treatments (e.g., diuretics, beta-blockers, calcium-channel blockers, and ACE inhibitors) [11–13], but the quality of evidence were unclear. In order to establish the efficacy and safety of Chinese herbal medicine for treating primary hypertension, an overview is needed to (1) summarize the conclusions of these reviews, (2) evaluate the methodological quality of these reviews, and (3) rate the confidence in the effect on each outcome.

## Methods

### Search strategy

Systematic searches of the following electronic databases were conducted: PubMed (1950 to December 2015), Chinese Biomedical database (1980 to December 2015), China Knowledge Resource Integrated Database (1980 to December 2015), and Wanfang database (1998 to December 2015), Search strategies for PubMed, EMBASE, and Chinese Biomedical database consisted of relevant MeSH terms, which were adapted for the respective databases and are available on request. Text word “Chinese herbal medicine”, “traditional Chinese medicine” and “alternative medicine” were used to search target reviews. Only English and Chinese papers were included. An additional file shows more search strategy detail (see Additional file 1).

### Selection of reviews

We included systematic reviews that met the following criteria: (1) evaluated the effects of Chinese herbal medicine on primary hypertension compared with conventional drugs; (2) provided a clearly defined clinical question, inclusion and exclusion criteria, and searching strategies; and (3) summarized the results for at least one desired outcome. Systematic reviews that had insufficient information for data extraction, translations, and duplicates were excluded.

### Data extraction

Two reviewers (ZXK and FMX) independently extracted information from the included studies using a standard form. We used original study reports only if specific data were missing. We extracted the following information:

- Basic information including publication year, retrieval strategy, inclusion criteria, quality assessment methods, and conclusions
- Number of included studies and participants
- Drug used, dose, and formulation (if formulation was available)
- Outcomes (including desirable outcomes and adverse events)

### Assessment of methodological quality and quality of evidence

#### Methodological quality

We used the assessing the methodological quality of systematic reviews (AMSTAR) [14] scale to assess the methodological quality of the included reviews. Each review was assessed by two researchers (ZXK and FMX) independently, and any disagreements were resolved by a third author (LYD). For each item, a judgment of “yes” or “no” was assigned according to judgment criteria of AMSTAR. An additional file provides the criterion to score methodological quality of systematic reviews (see Additional file 2). The number of “yes” will be counted as the total score of AMSTAR, which can reflect the overall methodological quality of reviews. If the total score is less than seven, which indicates the overall methodological quality of review is poor. The assessment process was based on the following 11 items:

- Was a priori design provided?
- Was there duplicate study selection and data extraction?
- Was a comprehensive literature search performed?
- Were published and unpublished studies included irrespective of language of publication?
- Was a list of studies (included and excluded) provided?
- Were the characteristics of the included studies provided?
- Was the scientific quality of the included studies assessed and documented?
- Was the scientific quality of the included studies used appropriately in formulating conclusions?
- Were the methods used to combine the findings of studies appropriate?
- Was the likelihood of publication bias assessed?
- Was a conflict of interest stated?

#### Quality of evidence

The quality of evidence reflects the extent to which confidence in an estimate of the effect is adequate to support a particular recommendation [15]. The quality of evidence for each outcome was rated following the Grading of Recommendations, Assessment, Development, and Evaluation (GRADE) Handbook [15] by two reviewers (ZXK and FMX) independently, and disagreements were resolved by a third reviewer (LYD). GRADE classified the quality of evidence into four levels: high, moderate, low, and very low (Table 1) [15]. The rating process was based on the following five downgrading factors.

**Table 1:** Definition of the four levels of evidence by GRADE [15]

Quality level	Definition
High	We are very confident that the true effect lies close to that of the estimate of the effect
Moderate	We are moderately confident in the effect estimate: the true effect is likely to be close to the estimate of the effect, but there is a possibility that it is substantially different
Low	Our confidence in the effect estimate is limited: the true effect may be substantially different from the estimate of the effect
Very low	We have very little confidence in the effect estimate: the true effect is likely to be substantially different from the estimate of effect

i) Risk of bias was assessed on the basis of the methodological quality of RCTs included in the systematic reviews and considered allocation concealment, blinding, incomplete outcome data, selective reporting, and other factors [16, 17]. In this review, we rated the factor relied on the risk of bias assessments by the authors of the included reviews.
ii) Inconsistency (i.e., heterogeneity) was assessed according to the outcomes of the *χ* ^2^ test and *I* ^2^ statistic reported in the systematic reviews. If *I* ^2^ was >50 %, *P* < 0.05, and the heterogeneity could not be explained by conducting subgroup analysis or meta-regression, the quality of evidence was downgraded [18].
iii) Indirectness was defined as having an indirect comparison in one of the following four aspects: population, intervention, comparator, and outcome (PICO). These four aspects were judged depending on the target PICO of interest [19].
iv) Imprecision was assessed in different ways for different types of data. For dichotomous outcomes, the quality of evidence was downgraded if either of the following two conditions were true [20]: (1) the total number of events was less than 300, or (2) the 95 % confidence interval (CI) of pooled risk ratio/odds ratio included both 1 and either 0.75 or 1.25. For continuous outcomes, the reasons for downgrading were (1) total population size less than 400, or (2) the 95 % CI of pooled mean difference/weighted mean difference included 0 and either −0.5 or 0.5.
v) Publication bias was assessed through funnel plots and Egger’s test. A two-tailed *P* value of <0.05 was considered to indicate publication bias. When there are only few studies included in the systematic review, the publication bias is challenging to interpret by funnel plots or statistical tests. Under these circumstances, we assessed publication bias based on the search methodology, databases searched, whether filters had been used, and inclusion of unpublished studies and gray literature (conference abstracts, protocols, and books) [21].

For each downgrade factor, a judgment of “no”, “serious” (downgrade by one level), or “very serious” (downgrade by two levels) was assigned. At the very beginning, the quality of evidence of all outcomes were classified as “high” by default; after rating, each outcome received a quality level of high, moderate, low, or very low.

#### Data analysis

A narrative description of the included reviews was undertaken. We have tabulated review-level summaries for all the outcomes listed above from each of the included reviews. Where outcomes were meta-analyzed within a review, we extracted and reported pooled effect sizes. Where no quantitative pooling of effect sizes was reported, or where outcomes were reported descriptively by single studies, we reported these results using a standardized language indicating direction of effect and statistical significance. For continuous outcomes, we summarized data using the weighted mean difference (WMD) with 95 % confidence interval (CI) as reported in the included reviews. For dichotomous outcomes, we presented the risk ratio (RR) or odds ratio (OR) and 95 % CI as appropriate.

## Results

**Fig. 1:**
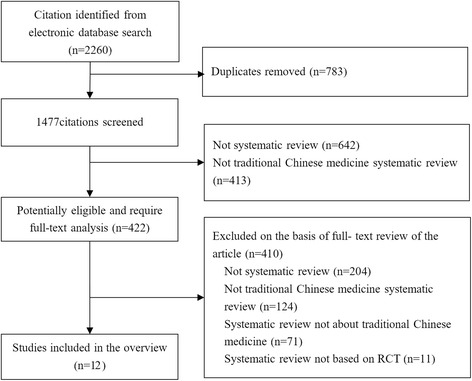
Flow diagram showing the search process and study selection

A total of 2260 records yielded from electronic databases. After removing duplicates, 1477 studies were screened by the titles or abstracts and 422 studies were assessed through the full texts. Finally, 12 systematic reviews about Chinese herbal medicine for primary hypertension were included in this overview [11–13, 22–30] (Fig. 1).

### Description of the included reviews

Among the 12 included reviews, all the reviews were published in Chinese, the publication time ranged from 2006 to 2014, and 83 % [11–13, 22–26, 29, 30] were published in the recent 5 years. Four reviews [11, 12, 22, 23] reported the age of the patients, and one [23] reported the follow-up time. Only one review compared Chinese herbal medicine with conventional drugs [13]; the others compared Chinese herbal medicine combined with conventional drugs against single conventional drugs. Nine reviews [13, 22–28, 30] adopted the Jadad scale, and three [21, 23, 24] used risk of bias tools [31] to assess the methodological quality of RCTs included in the review. Table 2 shows the characteristics of included systematic reviews.

**Table 2:** Characteristics of the included systematic reviews

Researchers and publication time	Types of included studies	Number of studies (total sample)	Age	Interventions		Follow-up time	Evaluation criteria of methodology	Conclusions
				Treatment group	Control group			
Chen 2014 [11]	RCT	12 (1001)	39–80	Banxia baizhu tianma tang + conventional medicine	Conventional medicine	–	Jadad	As compared with conventional medicine banxia baizhu tianma tang combined with it can improve the clinical curative effect in treating high blood pressure
Wang 2012 [12]	RCT	11 (981)	36–86	Danshen injection + control group	Hemostatic agents、dehydrator, regulation of blood pressure, brain cell protective agent	6 months	Jadad	Danshen injection has a certain treatment effect in treating hypertensive cerebral hemorrhage, and the earlier the better
Guo 2013 [13]	RCT	14 (1364)	–	Liuwei dihuang pill + conventional medicine	Conventional medicine	–	Jadad	The whole therapy effect of liuwei dihuang pill combined with conventional medicine is better than that of single conventional medicine, and so do the SBP and DBP
Zhou 2012 [22]	RCT	8 (554)	–	Tianma gouteng yin + captopril	Captopril	–	Jadad	Compared with single captopril, the clinical efficacy of tianma gouteng yin combined with captopril is more better, which can improve symptoms with better blood pressure effect
Dong 2011 [23]	RCT	6 (543)	–	Tianma gouteng yin + captopril	Captopril	–	Jadad	Tianma gouteng yin may obtain better treatment result and more security than enalapril in treatment of essential hypertension
Ren 2006 [24]	RCT	11 (1010)	–	TCM combination therapy including Chinese herb medicine, Chinese patent medicines, acupuncture, etc.) + conventional medicine	Conventional medicine	–	Jadad	Traditional Chinese medicine may have similar effect with conventional medicine in primary hypertension therapy
Dai 2010 [25]	RCT	9 (655)	–	Therapied by Chinese herb medicine or combined with conventional medicine	Conventional medicine	–	Jadad	Traditional Chinese medicine can reduce the SBP and DBP effectively, improve efficiency, integrated Chinese and Western treatment is more better
Du 2014 [26]	RCT	10 (1777)	–	Yangxue qingnao granules + conventional medicine	Conventional medicine	–	Risk of bias	Yangxue qingnao granules can significantly improve headache, dizziness, insomnia symptoms of high blood pressure
Li 2012 [27]	RCT	17 (1323)	–	TCM combination therapy including Chinese herb medicine compound, Chinese patent medicines, acupuncture, etc.) + conventional medicine	Conventional medicine	–	Jadad	Traditional Chinese medicine has a certain effect in treatment of elderly hypertension patients and reduced pulse pressure with symptoms reduced
Xiong 2012 [28]	RCT	16 (1424)	19–78	Banxia baizhu tianma tang + blood pressure drugs	Blood pressure drugs or placebo	–	Risk of bias	Banxia baizhu tianma tang has better antihypertensive effect
Wang 2013 [29]	RCT	22 (1808)	30–74	Tianma gouteng yin + blood pressure drugs	Blood pressure drugs	–	Risk of bias	The efficacy and safety evidence of tianma gouteng yin, as an adjunct of blood pressure medicine, needs further study
Wu 2013 [30]	RCT	9 (784)	–	Tianma gouteng yin	Conventional medicine	–	Jadad	Tianma gouteng yin can effectively lower the SBP and DBP

“–”means do not report related information

### Methodological quality of the included reviews

AMSTAR scale was used to evaluate the methodological quality of the included reviews. All of the included reviews were not registered [11–13, 22–30] in advance. Five reviews [24, 25, 27, 28, 30] did not provide the search strategies, which could not respect the process of the literature selection and data extraction. Five reviews [23–27] did not search gray literature, two reviews [27, 29] did not provide information of the included and excluded articles, and six reviews [24–27, 29, 30] did not provide the basic information of the included articles. Additionally, one review [30] did not appropriately explain the findings of studies, four reviews [11–13, 28, 30] did not assess for publication bias, and nine studies [22–30] did not state the conflicts of interest. Table 3 shows the methodological quality of the included studies.

**Table 3:** AMSTAR for methodological quality of included systematic reviews

Included studies	Item 1	Item 2	Item 3	Item 4	Item 5	Item 6	Item 7	Item 8	Item 9	Item 10	Item 11	Total score
Chen 2014 [11]	N	Y	Y	Y	Y	Y	Y	Y	Y	Y	N	9
Wang 2012 [12]	N	Y	Y	N	Y	Y	Y	Y	Y	Y	N	8
Guo 2013 [13]	N	N	Y	N	Y	N	Y	Y	Y	Y	N	6
Zhou 2012 [22]	N	N	Y	N	Y	N	Y	Y	Y	Y	N	6
Dong 2011 [23]	N	Y	Y	N	Y	N	Y	Y	Y	Y	N	7
Ren 2006 [24]	N	N	N	N	N	N	Y	Y	Y	Y	N	4
Dai 2010 [25]	N	N	Y	Y	Y	Y	Y	Y	Y	N	N	7
Du 2014 [26]	N	Y	N	Y	N	N	Y	Y	Y	Y	N	6
Li 2012 [27]	N	N	N	Y	Y	N	Y	N	Y	Y	N	5
Xiong 2012 [28]	N	Y	Y	Y	Y	Y	Y	Y	Y	N	Y	9
Wang 2013 [29]	N	Y	Y	Y	Y	Y	Y	Y	Y	N	Y	9
Wu 2013 [30]	N	Y	Y	Y	Y	Y	Y	Y	Y	N	Y	9

Y means adequate; N means inadequate. Item 1. Was an a priori design provided? Item 2. Was there duplicate study selection and data extraction? Item 3. Was a comprehensive literature search performed? Item 4. Were published and unpublished studies included irrespective of language of publication? Item 5. Was a list of studies (included and excluded) provided? Item 6. Were the characteristics of the included studies provided? Item 7. Was the scientific quality of the included studies assessed and documented? Item 8. Was the scientific quality of the included studies used appropriately in formulating conclusions? Item 9. Were the methods used to combine the findings of studies appropriate? Item 10. Was the likelihood of publication bias assessed? Item 11. Was a conflict of interest stated?

### Effect of interventions

#### Antihypertensive effect

**Fig. 2:**
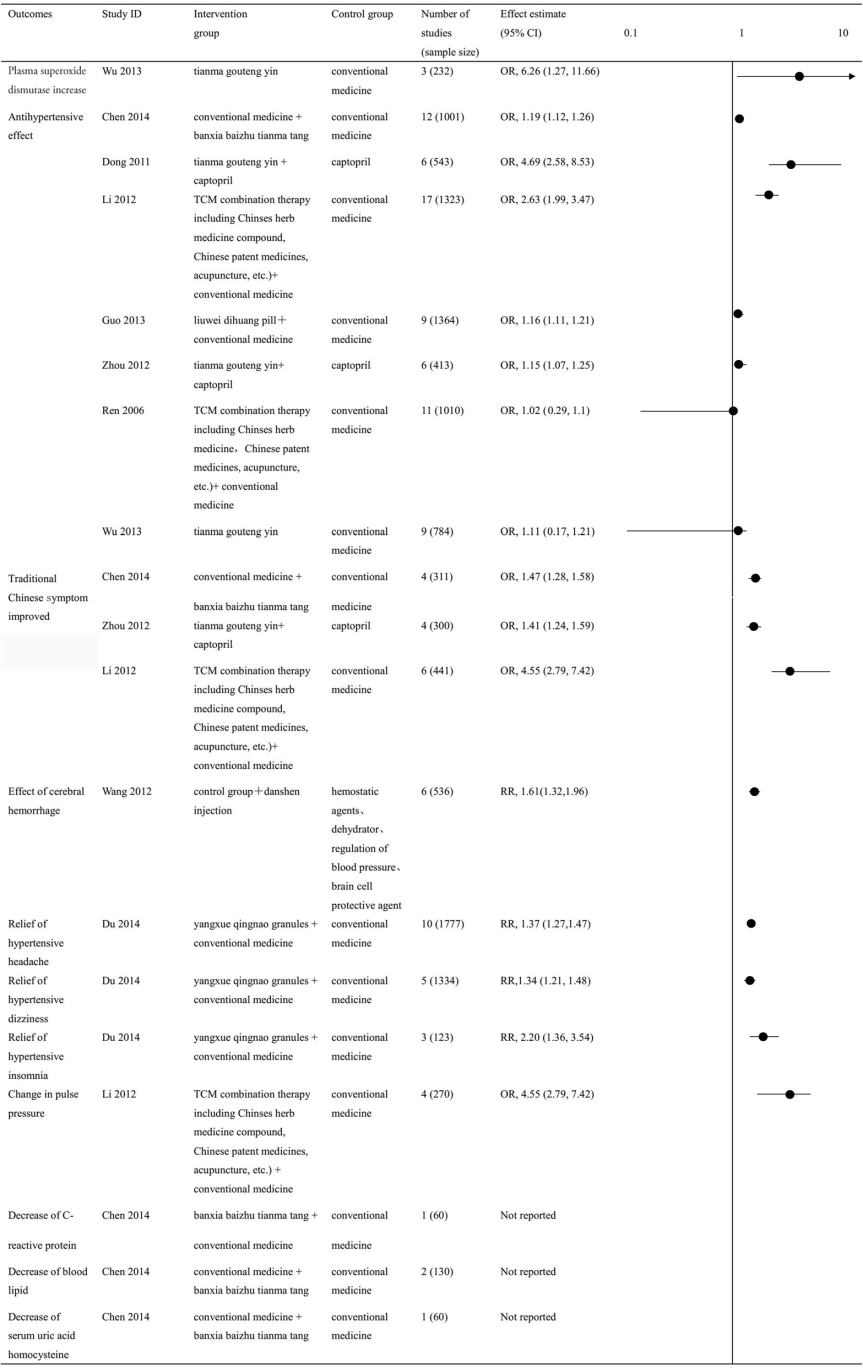
Summary of findings for dichotomous outcomes

Seven reviews [11, 13, 22–24, 27, 30] analyzed the antihypertensive effect of Chinese herbal medicines on treating primary hypertension; among which five reviews [22, 24–26, 30] indicated that Chinese herbal medicine combined with the conventional medicine is better than the single conventional medicine, and the difference was statistically significant (*P* < 0.05). The combination of Chinese herbal medicines and conventional medicine including banxia baishu tianma tang plus conventional medicine [11] (OR = 1.19 [1.12, 1.26], moderate quality of evidence), liuwei dihuang pill plus conventional medicine [13] (OR = 1.16 [1.11, 1.21], moderate quality of evidence), tianma goutengyin plus captopril [23] (OR = 4.69 [2.58, 8.53], low quality of evidence), and acupuncture plus conventional medicine [27] (OR = 2.63 [1.99, 3.47], low quality of evidence). See Fig. 2 for more information.

#### Decreasing SBP and DBP effect

**Fig. 3:**
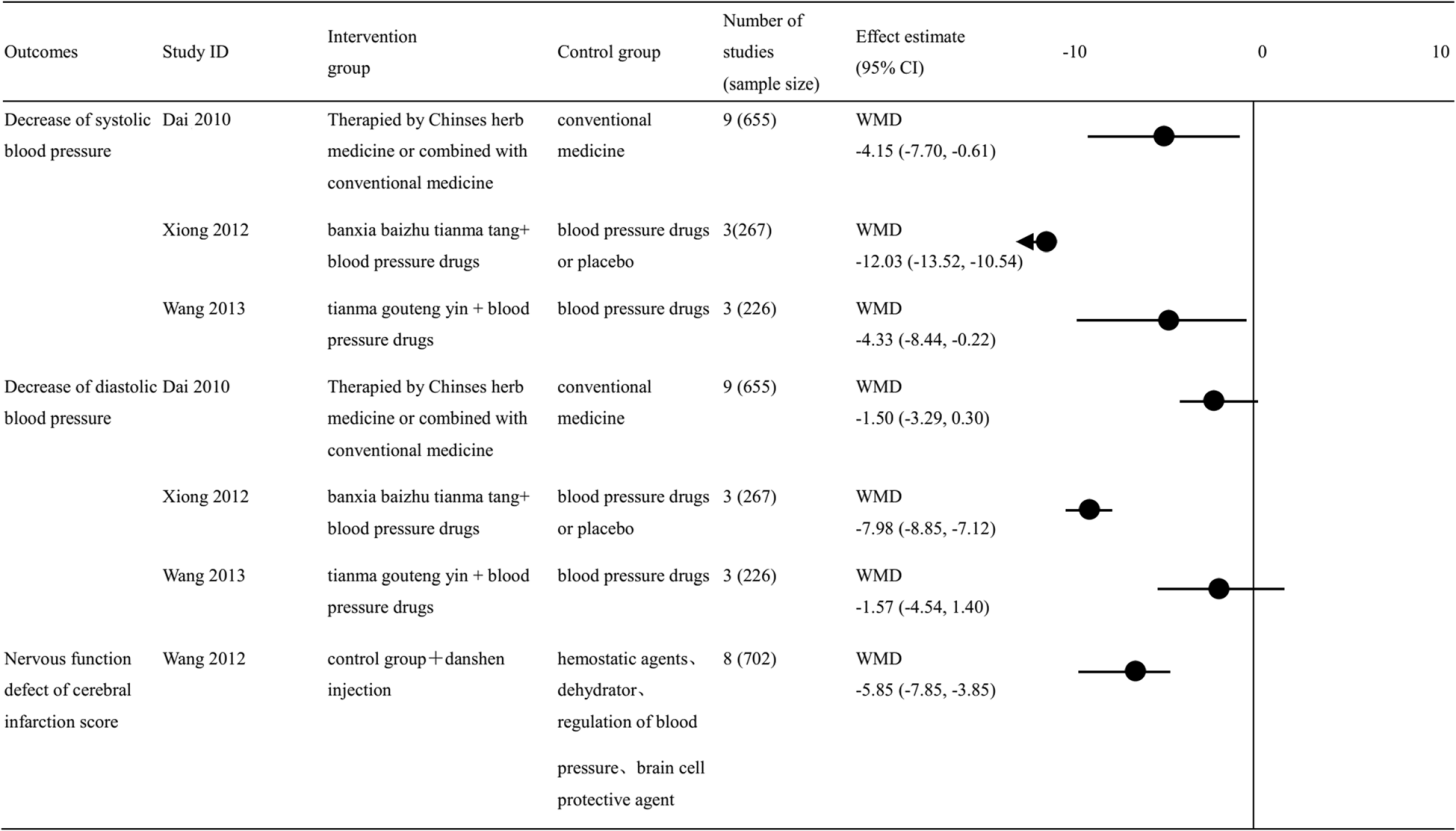
Summary of findings for continuous outcomes

Three reviews [25, 28, 29] analyzed the changes of SBP and DBP levels in patients using Chinese herbal medicines to treat primary hypertension. All the three reviews showed that traditional Chinese medicine combined with conventional medicine were more efficient than single conventional medicine on the effect of SBP. The combination of Chinese herbal medicines and conventional medicine can decease SBP including Chinses herb medicine plus conventional medicine [25] (WMD = −4.15 [−7.70, −0.61], low quality of evidence), banxia baizhu tianma tang plus blood pressure drugs [28] (WMD = −12.3 [−13.52, −10.54], low quality of evidence), and tianma goutengyin plus blood pressure drugs [29] (WMD = −4.33 [−8.44, −0.22], low quality of evidence). However, only one review [28] showed a beneficial result for DBP [WMD, −7.98 (−8.85, −7.12), low quality of evidence] (Fig. 3).

#### Traditional Chinese symptom improvement

Three reviews [11, 22, 27] analyzed the traditional Chinese symptom improvement. All reviews showed that banxia baizhu tianma tang (OR = 1.47 [1.28, 1.58], low quality of evidence), tianma gouteng yin (OR = 1.41 [1.24, 1.59], low quality of evidence), and acupuncture (OR = 4.55 [2.79, 7.42], low quality of evidence), combined with conventional medicine were better than single conventional medicine for traditional Chinese symptom improvement; all differences were statistically significant (*P* < 0.05) (Fig. 2).

#### Adverse events

Five reviews [11, 12, 22, 23, 28] evaluated adverse events associated with Chinese herbal medicine combined with conventional medicine, including headaches, swelling, heart palpitations, and lethargy. Two reviews [23, 28] reported that Chinese herbal medicine (tianma gouteng yin and banxia baizhu tianma tang) combined with conventional medicine for the treatment of primary hypertension was safe, without any adverse reactions noted.

#### Summary of other findings

One review [26] indicated that yangxue qingnao granules combined with conventional medicine had a better effect on hypertensive headache (RR = 1.37 [1.27, 1.47]), hypertensive dizziness (RR = 1.34 [1.21, 1.48]), and hypertensive insomnia (RR = 2.20 [1.36, 3.54]) than conventional medicine. Another review [11] compared banxia baizhu tianma tang plus conventional medicine with conventional medicine alone; however, they did not report the effects on C-reactive protein, blood lipid, serum uric acid, or homocysteine levels. This indicates that a selective reporting bias existed in this review.

#### Summary of quality of evidence

A total of 31 outcomes were measured by the 12 included reviews [11–13, 22–30]. Among these outcomes, the quality of evidence was high in none (0 %), moderate in three (10 %), low in 19 (61 %), and very low in nine (29 %). Of the five downgrading factors, the risk of bias (*n* = 31, 100 %) was the most common downgrading factor in the included reviews, followed by imprecision (*n* = 13, 42 %), inconsistency (*n* = 12, 39 %), publication bias (*n* = 12, 39 %), and indirectness (*n* = 0, 0 %). According to GRADE, the risk of bias is defined as a defect in random sequence generation, allocation concealment, blinding, incomplete outcome data, selective reporting, and other bias. Among these, random sequence generation was the most important factor contributing to the overall poor quality for these reviews. Table 4 shows the quality of evidence of the included reviews.

**Table 4:** GRADE for quality of evidence profile

Study ID	Outcomes (number of studies)	Risk of bias	Inconsistency	Indirectness	Imprecision	Publication bias	Quality of evidence
Chen 2014 [11]	Overall antihypertensive effect (12)	Serious^a^	No serious	No serious	No serious	Strongly suspected^b^	Low
	Traditional Chinese symptom improved (4)	Serious^a^	Serious^c^	No serious	No serious	Undetected	Low
	Decrease of C-reactive protein (1)	Serious^a^	No serious	No serious	Serious^d^	Strongly suspected^b^	Very low
	Decrease of blood lipid (2)	Serious^a^	Serious^c^	No serious	Serious^d^	Undetected	Very low
	Decrease of serum uric acid homocysteine (2)	Serious^a^	No serious	No serious	Serious^d^	Strongly suspected^e^	Very low
	Adverse events (1)	Serious^a^	No serious	No serious	Serious^d^	Strongly suspected^e^	Very low
Wang 2012 [12]	Effect of cerebral hemorrhage (6)	Serious^a^	No serious	No serious	No serious	Undetected	Moderate
	Nervous function defect of cerebral infarction score (8)	Serious^a^	Serious^c^	No serious	No serious	Undetected	Low
	Adverse events (5)	Serious^a^	Serious^c^	No serious	No serious	Undetected	Low
Guo 2013 [13]	Overall antihypertensive effect (9)	Serious^a^	No serious	No serious	No serious	Undetected	Moderate
Zhou 2012 [22]	Overall antihypertensive effect (6)	Serious^a^	No serious	No serious	No serious	Undetected	Moderate
	Traditional Chinese symptom improved (4)	Serious^a^	Serious^c^	No serious	No serious	Undetected	Low
	safety (5)	Serious^a^	Serious^c^	No serious	No serious	Undetected	Low
Dong 2011 [23]	Overall antihypertensive effect (6)	Serious^a^	No serious	No serious	No serious	Strongly suspected^b^	Low
	safety (2)	Serious^a^	No serious	No serious	Serious^d^	Strongly suspected^e^	Very low
Ren 2006 [24]	Overall antihypertensive effect (11)	Serious^a^	No serious	No serious	No serious	Strongly suspected^b^	Low
Dai 2010 [25]	Decrease of systolic blood pressure (9)	Serious^a^	No serious	No serious	Serious^d^	Undetected	Low
	Decrease of diastolic blood pressure (9)	Serious^a^	No serious	No serious	Serious^d^	Undetected	Low
Du 2014 [26]	Relief of hypertensive headache (10)	Serious^a^	No serious	No serious	No serious	Strongly suspected^b^	Low
	Relief of hypertensive dizziness (5)	Serious^a^	No serious	No serious	No serious	Strongly suspected^b^	Low
	Relief of hypertensive insomnia (3)	Serious^a^	No serious	No serious	Serious^d^	Strongly suspected^b^	Very low
Li 2012 [27]	Overall antihypertensive effect (17)	Serious^a^	No serious	No serious	No serious	Strongly suspected^b^	Low
	Traditional Chinese symptom improved (6)	Serious^a^	No serious	No serious	No serious	Strongly suspected^b^	Low
	Changes of pulse pressure (4)	Serious^a^	Serious^c^	No serious	Serious^d^	Undetected	Very low
Xiong 2012 [28]	Decrease of systolic blood pressure (3)	Serious^a^	Serious^c^	No serious	Serious^d^	Undetected	Very low
	Decrease of diastolic blood pressure (3)	Serious^a^	Serious^c^	No serious	Serious^d^	Undetected	Very low
	Adverse events (4)	Serious^a^	No serious	No serious	Serious^d^	Undetected	Low
Wang 2013 [29]	Decrease of systolic blood pressure (3)	Serious^a^	Serious^c^	No serious	No serious	Undetected	Low
	Decrease of diastolic blood pressure (3)	Serious^a^	Serious^c^	No serious	No serious	Undetected	Low
Wu 2013 [30]	Plasma superoxide dismutase increase (3)	Serious^a^	No serious	No serious	Serious^d^	Undetected	Low
	Overall antihypertensive effect (9)	Serious^a^	Serious^c^	No serious	No serious	Undetected	Low

^a^Unclear random sequence generation, allocation concealment blinding not done in all studies

^b^Statistical test for publication bias was underpowered

^c^ *I* ^2^ >50 %

^d^Insufficient sample size and wide confidence interval

^e^Incomplete retrieval for unpublished studies and gray literature

## Discussion

Although the systematic review is one of the most important research methods and provides the strongest level of evidence in evidence-based medicine [32], only those reviews with qualified methodologies and a high quality of evidence can provide comprehensive and reliable evidence to decisionmakers [33]; otherwise, review findings are likely to mislead decisionmakers. An overview of systematic reviews is a comprehensive evaluation method, which summarizes the findings, detects the methodological quality, and grades the evidence quality of all systematic reviews on one disease. In this overview, almost 60 % of the systematic reviews were found to have a good methodology quality (AMSTAR score ≥7). A summary of the findings of these reviews showed that Chinese herbal medicine combined with conventional medicine in the treatment of primary hypertension has better efficacy and safety than treatment with a single conventional medicine. This finding might reflect that Chinese herbal medicine combined with conventional medicine can improve the clinical symptoms and delay disease progression in patients with primary hypertension. Additionally, Chinese herbal medicine combined with conventional medicine offers the potential to reduce side effects and medical costs when compared with single conventional medicine.

However, we found that 90 % of the outcomes were of low or very low quality of evidence when using the GRADE criteria to evaluate the systematic reviews, indicating that the true effect might be substantially different from the effect estimated in these reviews. Of the five downgrading factors, the risk of bias was the most common factor downgrading the level of evidence. All of the outcomes from the 12 reviews were downgraded for this factor, and failure of random sequence generation was the most important factor contributing to the overall poor risk of bias scores. This indicates that rigorous training on conducting Chinese herbal medicine trials for investigators is warranted. Imprecision was downgraded most often due to insufficient sample size, while inconsistency was downgraded due to unreasonable inclusion criteria and large *I* ^2^ squared values. Finally, downgrading of evidence for publication bias was most commonly due to not reviewing gray literature and presenting underpowered statistical tests.

Most of the outcomes in the systematic reviews of Chinese herbal medicine for primary hypertension were surrogate outcomes, such as blood pressure and nervous function defect score. These outcomes do not reflect all effects of the complex pathological process associated with primary hypertension [33] or substitute for the measurement of end-outcomes such as mortality, end-organ damage, stroke, coronary artery disease, and renal failure. Sometimes, advantages might outweigh the disadvantages when we use surrogate outcomes to measure the effectiveness of an intervention. For example, clofibrate, a fibrate lipid-lowering drug for ischemic heart disease patients, could reduce the risk of ischemic heart disease in patients by 20 %, but the all-cause mortality increased to 44 % [34]. Therefore, future studies assessing the use of Chinese herbal medicine in treatment of primary hypertension need to be conducted with a focus on end outcomes.

This overview has several strengths: we used a structured and explicit approach, a comprehensive search strategy, and eligibility criteria designed to identify systematic reviews about the use of Chinese herbal medicine for the treatment of primary hypertension. We also created strict quality assessment criteria to evaluate the methodological quality and the quality of evidence for each review, which increases the validity and reliability of the findings. We used the GRADE system, a previously validated scientific approach, to rate the quality of the evidence. This overview, however, also has some limitations: we excluded systematic reviews that had insufficient information for extracting data, which might introduce selection bias. Publication bias was also sometimes challenging to assess with funnel plots and Egger’s test. For instance, although the formal statistical tests showed no significant publication bias, these tests might have been severely underpowered given the small number of original studies in the systematic reviews. Some systematic reviews using the fixed effect model resulted in a large *I* ^2^ values (more than 50 %), which were incorrect. Finally, some of the reviews’ authors might desire to compare Chinese herbal medicine versus drugs, and some of the authors were Chinese medical workers, so it is possibility exist interpretation bias in some reviews.

## Conclusions

Physicians should be cautious when applying the interventions in these reviews for primary hypertension patients in clinical practice. Our overview suggests that the methodological quality and quality of evidence in Chinese herbal medicine for primary hypertension is fairly poor. More efforts must be made to improve the quality of RCTs about Chinese herbal medicine. First, clinical trials about Chinese herbal medicine should be designed in high methodological quality, registered on the Chinese Clinical Trial Register (ChiCTR) platform [35], and reported following CONSORT checklist [36, 37] to minimize bias. Second, systematic reviews about Chinese herbal medicine should be conducted following the Cochrane Handbook for Systematic Reviews [31] to improve the methodological quality and report the systematic reviews according to Preferred Reporting Items for Systematic Reviews and Meta-Analyses (PRISMA) statement; Third, Chinese GRADE Center should make a further effort to spread the GRADE system and train guideline developers on how to make recommendations based on low and very low quality evidence [38].

## Additional files

Additional file 1: Search strategy. The process of how to search PubMed, EMBASE, Cochrane library, CBM, CNKI, and Wang fang database was given in this file. (PDF 180 KB)
Additional file 2: Judgment criteria of AMSTAR. This file provided a criterion of how to score the methodological quality of systematic reviews. (PDF 165 KB)

## Abbreviations

ACE: Angiotensin-converting enzyme
AMSTAR: Assessing the Methodological Quality of Systematic Reviews
CBM: Chinese Biomedical database
ChiCTR: Chinese Clinical Trial Register
CI: Confidence interval
CNKI: China Knowledge Resource Integrated Database
CVD: Cardiovascular disease
DBP: Diastolic blood pressure
GRADE: Grading of Recommendations, Assessment, Development, and Evaluation
PRISMA: Preferred Reporting Items for Systematic Reviews and Meta-Analyses
RCT: Randomized controlled trials
SBP: Systolic blood pressure
