# Supplementary material for: Chinese herbal medicine for the treatment of primary hypertension: a methodology overview of systematic reviews
Source: Syst Rev. 2016 Oct 20;5:180. doi: 10.1186/s13643-016-0353-y (PMC5072301; doi:10.1186/s13643-016-0353-y)
Supplement: Additional file 1: — Search strategy. The process of how to search PubMed, EMBASE, Cochrane library, CBM, CNKI, and Wang fang database was given in this file. (PDF 180 KB) [file 13643_2016_353_MOESM1_ESM.pdf]

Pubmed
#1 "Hypertension"[Mesh]
#2 "Cochrane database of systematic reviews"[Journal]
#3 search[Text Word]
#4 meta-analysis[Publication Type]
#5 Medline[Text Word]
#6 systematic review[Text Word]
#7 #2 OR #3 OR #4 OR #5 OR #6
#8 "Medicine, Chinese Traditional"[Mesh]
#9 "Complementary Therapies"[Mesh]
#10 #9 OR ##8
#11 #10 AND #7 AND #1

Embase：
#1 'Medicine, Chinese Traditional'/exp
#2 'alternative medicine'/exp
#3 #1 OR #2
#4'meta analysis'/exp
#5 review.pt.
#6 search.tw.
#7 #4 OR #5 OR #6
#8 'hypertension'/exp
#9 #3 AND #7 AND #8

Cochrane Library
#1 MeSH descriptor: [Medicine, Chinese Traditional] explode all trees
#2 MeSH descriptor: [Complementary Therapies] explode all trees
#3 #1 OR #2
#4 MeSH descriptor: [Hypertension] explode all trees
#5 #3 AND #4

CBM
#1 "Meta 分析"[不加权:扩展]
#2 "系统评价"[常用字段:智能]
#3 "荟萃分析"[常用字段:智能]
#4 #1 OR #2 OR #3
#5 "高血压"[不加权:扩展]
#6 #5 AND #4

CNKI
#1 主题 meta
#2 主题 荟萃分析

#3 主题 系统评价
#4 #1 OR #2 OR #3
#5 高血压
#6 #4 AND #5

Wanfang
#1 主题 meta
#2 主题 荟萃分析
#3 主题 系统评价
#4 #1 OR #2 OR #3
#5 高血压
#6 #4 AND #5
